# The Reviewer to Dr. Yeats

**Published:** 1816-06

**Authors:** 


### The REVIEWER to Dr. YEATS.

THE Reviewer of Dr. Yeats's letter on Hydrencephalus, feels much gratified at having given *general* satisfaction to that intelligent physician in the analysis of his work. Dr. Yeats's communication in the last Number of this Journal shews, that the Reviewer misunderstood the passage relative to purgatives *combined* with mercury in hydrencephalus:—But the Reviewer begs leave to assure Dr. Yeats, that he has quite mistaken the passage respecting *local blood-letting* in page 311 of the Journal. The propriety of this measure was urged as *the sentiment of Dr. Yeats*, and reference was made to Dr. Parry merely as corroborative, or rather as elucidatory, of Dr. Yeats's opinion. This may be seen by the extract immediately afterwards introduced—"It is on *this principle* that *topical* abstractions of blood are so useful," &c. In extended analyses, such as the Medico-Chirurgical Journal is accustomed to give, the *sentiments* of the author must frequently be condensed, and conveyed in the *words* of the Reviewer, the latter only manifesting himself in his own shape occasionally, according to the best of his judgment.

---

Mr. Johnson in reply to Dr. Yeats's Postscript, can only say, that every circumstance in the Case of Hydrencephalus examined by him,* shewed the epigastric viscera as exhibiting the *first alarm* of cerebral effusion. From the length of time required for the cerebral dissection, the abdomen could not be examined; but Mr. Johnson does not think this of much consequence, since he has often observed great *functional* derangement prevail, without any *organic* disease. His opinion of the case alluded to is, that the fall occasioned some cerebral commotion which gave origin to the derangement of the digestive organs,

---

* Journal, page 367.

and that the *latter* ultimately occasioned effusion in the ventricles of the brain. Mr. Johnson is happy to find that a coincidence of opinion respecting the *portal circulation* prevails between Dr. Yeats and himself. When two people hit on a new and a similar idea, without any intercourse, it is a strong proof of its being founded in truth.

